# Method Matters: Effect of Purification Technology on Neutrophil Phenotype and Function

**DOI:** 10.3389/fimmu.2022.820058

**Published:** 2022-02-10

**Authors:** Marfa Blanter, Seppe Cambier, Mirre De Bondt, Lotte Vanbrabant, Noëmie Pörtner, Sara Abouelasrar Salama, Mieke Metzemaekers, Pedro Elias Marques, Sofie Struyf, Paul Proost, Mieke Gouwy

**Affiliations:** Laboratory of Molecular Immunology, Department of Microbiology, Immunology and Transplantation, Rega Institute, KU Leuven, Leuven, Belgium

**Keywords:** neutrophil activation, immunomagnetic separation, density-gradient centrifugation, migration, degranulation, phagocytosis, NETosis, respiratory burst

## Abstract

Neutrophils are the most abundant leukocytes in human blood and the first cells responding to infection and injury. Due to their limited *ex vivo* lifespan and the impossibility to cryopreserve or expand them *in vitro*, neutrophils need to be purified from fresh blood for immediate use in experiments. Importantly, neutrophil purification methods may artificially modify the phenotype and functional characteristics of the isolated cells. The aim of this study was to expose the effects of ‘classical’ density-gradient purification *versus* the more expensive but faster immunomagnetic isolation on neutrophil phenotype and functionality. We found that in the absence of inflammatory stimuli, density-gradient-derived neutrophils showed increased polarization responses as well as enhanced release of reactive oxygen species (ROS), neutrophil extracellular traps (NETs) and granular proteins compared to cells derived from immunomagnetic isolation, which yields mostly quiescent neutrophils. Upon exposure to pro-inflammatory mediators, immunomagnetic isolation-derived neutrophils were significantly more responsive in polarization, ROS production, phagocytosis, NETosis and degranulation assays, in comparison to density-gradient-derived cells. We found no difference in chemotactic response in Multiscreen and under-agarose migration assays, but Boyden assays showed reduced chemotaxis of immunomagnetic isolation-derived neutrophils. Finally, we confirmed that density-gradient purification induces artificial activation of neutrophils, evidenced by *e.g.* higher expression of CD66b, formyl peptide receptor 1 (FPR1) and CD35, and the appearance of a separate neutrophil population expressing surface molecules atypical for neutrophils (*e.g.* CXCR3, MHC-II and CD14). Based on these results, we recommend using immunomagnetic separation of neutrophils for studying neutrophil polarization, phagocytosis, ROS production, degranulation and NETosis, whereas for Boyden chemotaxis assays, the density-gradient purification is more suitable.

## 1 Introduction

Neutrophilic granulocytes, or neutrophils, constitute the majority (50-70%) of circulating human leukocytes (1). These innate immune cells have a broad variety of effector functions and are the first responding cells during inflammation and infection. Under physiological conditions, neutrophils circulate in a resting state to patrol the body for pathogens, being indispensable for the initiation, modulation and resolution of inflammation. The presence of an inflammatory or infectious trigger induces priming and activation of neutrophils. This process is tightly regulated, as activated neutrophils release toxic content, which can be detrimental to healthy tissue. Upon appropriate stimulation, neutrophils initiate the process of rolling and adhesion to the vessel wall, followed by transmigration into the inflamed tissue where they exert their effector functions (2, 3). These functions include phagocytosis, production of reactive oxygen species (ROS), release of pre-synthesized proteins (*e.g.* defensins) and enzymes (*e.g.* myeloperoxidase [MPO], neutrophil elastase [NE] and matrix metalloproteinases [MMPs]), release of neutrophil extracellular traps (NETs) and the production of cytokines (*e.g.* interleukin-1β [IL-1β], tumor necrosis factor-α [TNF-α]) and chemokines (4). After eliminating the potentially harmful agent, neutrophils either undergo apoptosis and clearance by local macrophages or return to the bone marrow (5, 6). Circulating blood neutrophils that are not recruited to tissue are believed to have a limited lifespan of only 7-10 hours, whereas neutrophils in inflamed tissues may live up to 7 days (7).

To study neutrophils *ex vivo*, the cells should ideally resemble the circulating, quiescent population found in human blood. However, as neutrophils are terminally differentiated cells, they cannot be expanded *in vitro* nor successfully cryopreserved, making them challenging to use in experiments (8). Therefore, the most valid approach for studying human neutrophils in their most physiological state is the use of freshly isolated cells from peripheral blood samples. Following blood collection, neutrophils should be purified as soon as possible (within 1 hour after collection) to prevent artificial activation and apoptosis (9). To date, several methods have been established to isolate neutrophils from whole blood samples. However, the isolation procedure may significantly influence the quantity and quality of the purified cells. The oldest established method by Boyum et al. (10) relies on density-gradient purification, in which blood is pipetted on top of a polysaccharide solution (*e.g.* Pancoll, Ficoll) and subsequently centrifuged. This leads to the formation of a gradient, with peripheral blood mononuclear cells (PBMCs) resting on top of the polysaccharide layer while the granulocytes sediment together with the erythrocytes. After removing the PBMC layer, the cells are subjected to a dextran treatment (sedimenting the majority of erythrocytes) and subsequently exposed to a hypotonic shock (for lysis of residual erythrocytes) (10). Over time, new methods have been developed such as fluorescence-activated cell sorting (FACS). With this technique, cells are selected based on their size, morphology and protein expression, which allows the separation of different cell types from a mixture. However, this method is time-consuming and expensive, and not ideal for neutrophils due to the activation induced by cross-linking of surface receptors with antibodies (11). The most recently established technique is immunomagnetic selection using magnetic beads coupled to antibodies against non-neutrophil lineage markers. This technique is less time-consuming (approximately 1 hour of purification compared to *circa* 3 hours needed for density-gradient purification) but is more expensive compared to the standard density-gradient purification. Moreover, comparing studies that use different neutrophil isolation methods may be challenging, as research suggests that cells isolated with different methods may have a distinct activation state (12, 13). Nowadays, this is especially important since a variety of isolation methods are being used by different research groups.

The aim of this study was to dismantle and compare the effects of density-gradient or immunomagnetic purification methods on neutrophil phenotype and function. We found that neutrophils purified by immunomagnetic beads generally possess a lower activation state and respond more potently to activating stimuli in a variety of functional assays, compared to density-gradient-derived neutrophils. This shows that the purification method is a significant confounding factor. Our findings underline the importance of choosing the correct methodology for the unbiased study of neutrophils *in vitro.*

## 2 Materials And Methods

### 2.1 Reagents

Recombinant human CXCL8(6-77), TNF-α, interferon-γ (IFN-γ), IL-1β and granulocyte-macrophage colony-stimulating factor (GM-CSF) were purchased from Peprotech (Rocky Hill, NJ, USA). The bacterial tripeptide N-formyl-methionyl-leucyl-phenylalanine (fMLF), peptidoglycan from *Staphylococcus aureus* (PGN), phorbol 12-myristate 13-acetate (PMA), lipopolysaccharide (LPS) from *Klebsiella pneumoniae* and LPS from *Escherichia coli* were purchased from Sigma-Aldrich (St. Louis, MO, USA). CpG oligodeoxynucleotides were purchased from InvivoGen (San Diego, CA, USA).

### 2.2 Neutrophil Purification

Venous blood from healthy human volunteers was collected in EDTA-coated vacutainer tubes (BD Biosciences, Franklin Lakes, NJ, USA) and processed within 15 minutes after withdrawal.

#### 2.2.1 Density-Gradient Purification

Whole blood was diluted 1:2 in sterile Dulbecco′s Phosphate Buffered Saline (DPBS) without Ca^2+^ and Mg^2+^ and slowly pipetted on top of Pancoll (1.077 g/ml) (PAN-Biotech, Aidenbach, Germany) in a 2:3 volume ratio. The blood was spun down for 30 minutes at 400g without braking. Following centrifugation, the three top layers (*i.e.* diluted plasma, PBMCs and Pancoll) were carefully discarded, leaving a red pellet with erythrocytes and granulocytes. The pellet was then mixed with equal volumes of DPBS and 6% (*w/v*) dextran in DPBS, followed by incubation for 30 minutes at 37°C. Next, the supernatant containing neutrophils was transferred to a clean tube, and the cells were washed twice with DPBS (centrifugations were performed for 10 minutes at 177g). Subsequently, the cell pellet was suspended in 5 ml DPBS, and 25 ml sterile ultrapure water was added. After 30 seconds, 10 ml 3.6% (*w/v*) NaCl was added and mixed by inversion. The cells were spun down and washed with DPBS, whereupon a small aliquot was diluted in Turk’s solution (Sigma-Aldrich) and used to determine the cell concentration using a Bürker chamber. The average yield (± SD) was 1.72 × 10^6^ (± 0.66 × 10^6^) neutrophils/ml blood. The average purity (± SD) of the neutrophils (defined as live CD16^+^CD66b^+^ cells) was 95.2 (± 1.6) %.

#### 2.2.2 Immunomagnetic Isolation

Immunomagnetic isolation was performed using the EasySep Direct Human Neutrophil Isolation kit (StemCell Technologies, Vancouver, BC, Canada) according to the manufacturer’s instructions. Briefly, whole blood was transferred to 15 ml tubes (5 ml/tube) and supplemented with Isolation Cocktail (50 µl/ml of blood) and Rapidspheres (50 µl/ml of blood). After thorough mixing, the blood was incubated for 5 minutes at room temperature (RT). Subsequently, the mixture was diluted in DPBS without Ca^2+^ and Mg^2+^ (final volume of 12 ml/tube), and the tubes were transferred to an EasyEights magnet (StemCell Technologies). After incubation for 10 minutes at RT, the supernatant (containing plasma, neutrophils and a small percentage of contaminating cells) was carefully collected, transferred to a clean tube, supplemented with Rapidspheres (same volume as in the first step), mixed and incubated for 5 minutes at RT. The tube was placed back in the magnet and incubated for 5 minutes at RT. The neutrophils were carefully transferred to a clean tube and incubated for another 5 minutes in the magnet. Finally, the neutrophils were collected, spun down (8 minutes at 177g) and suspended in DPBS. A small aliquot of the cells was mixed with Turk’s solution and used to determine the cell concentration using a Bürker chamber. The average yield (± SD) was 1.75 × 10^6^ (± 0.51 × 10^6^) neutrophils/ml blood. The average purity (± SD) of the neutrophils (defined as live CD16^+^CD66b^+^ cells) was 97.3 (± 1.3) %.

### 2.3 Actin Polymerization Assay

The degree of actin polymerization in purified neutrophils was measured as previously described (14). Briefly, neutrophils were stimulated in suspension with CXCL8 (1-30 ng/ml) or fMLF (10^-9^-10^-7^ M) for 30 seconds in an uncoated U-bottom 96-well plate (1.5 × 10^6^ cells/ml [70 µl/well] in pre-warmed [37°C] RPMI-1640 + 1 mg/ml human serum albumin [HSA; Belgian Red Cross]). After stimulation, cells were placed on ice, fixed and permeabilized using the BD Cytofix/Cytoperm kit (BD Biosciences). Subsequently, the cells were resuspended in BD Perm/Wash buffer (BD Biosciences) containing Alexa Fluor 555-Phalloidin (2 U/ml; Invitrogen, Waltham, MA, USA), a dye which selectively stains filamentous (F)-actin. The cells were incubated for 20 minutes at 4°C, whereupon they were washed twice with BD Perm/Wash buffer and resuspended in flow cytometry buffer (PBS + 2% [*v/v*] fetal calf serum [FCS; Sigma-Aldrich] + 2 mM EDTA). The cellular F-actin content was quantified by flow cytometry using a BD LSRFortessa™ X-20 (BD Biosciences) equipped with DIVA software (BD Biosciences). FlowJo software (BD Biosciences, v.10.8.1) was used for downstream analysis.

### 2.4 Shape Change Assay

Shape change assays were performed to determine the responsiveness of suspended neutrophils to chemoattractants. Serial dilutions of CXCL8 (3-30 ng/ml) or fMLF (10^-9^-10^-7^ M) in shape change buffer (HBSS without Ca^2+^ and Mg^2+^, supplemented with 10 mM HEPES) were added to a flat-bottom 96-well plate (50 µl/well). Shape change buffer without chemoattractants served as negative control. Neutrophils (50 µl/well) were added to the plate at a concentration of 0.6 × 10^6^ cells/ml in prewarmed (37°C) shape change buffer. Following a stimulation period of 3 minutes, neutrophils were fixed with 100 µl of ice-cold 4% (*w/v*) formaldehyde in shape change buffer. One hundred cells per condition were counted microscopically and categorized as either activated (blebbed and elongated cells) or resting/not activated (round). The assessment was performed by two independent researchers, of whom at least one was blinded to the experimental conditions.

### 2.5 *In Vitro* Cell Migration Assays

The chemotactic response of purified neutrophils towards CXCL8 (3-150 ng/ml) or fMLF (10^-9^-10^-6^ M) was measured in three different *in vitro* chemotaxis assay systems, namely the Boyden chamber assay, the Multiscreen assay and the under-agarose assay. For the classical 48-well Boyden chamber technique, chemoattractants and neutrophils (1 × 10^6^ cells/ml) were suspended in Boyden chamber buffer (HBSS buffer enriched with 1 mg/ml HSA). Chemoattractants were added to the lower compartment of the Boyden chamber (30 µl/well) and were covered with a 5 µm pore size polyvinylpyrrolidone (PVP)-free polycarbonate membrane (GE Water & Process Technologies, Feasterville-Trevose, PA, USA). Buffer without chemoattractant was used as negative control. Neutrophils were added to the upper part of the chamber (50 µl/well) and migration was allowed during 45 minutes at 37°C. Subsequently, cells on the membranes were fixed and stained (Hemacolor Solution I–III, Merck, Kenilworth, NJ, USA). Migrated neutrophils were microscopically counted at the lower side of the membrane in 30 separate fields for each test condition. The directional migration of the neutrophils towards a chemoattractant is expressed as the chemotactic index (CI), which was calculated by dividing the total number of neutrophils migrated towards chemoattractant by the total number of cells migrated towards buffer.

The Multiscreen plate (Millipore Corporation, Billerica, MA, USA) is a disposable device with a 96-well filter plate (5 µm pore-size) and a 96-well receiver plate (15). Neutrophil cell migration through the 96-well filter plate occurs in response to a chemotactic gradient. The cell suspension (100 µl/well in the 96-well filter plate at a concentration of 2.5 x 10^6^ cells/ml) and chemoattractant dilutions (150 µl/well in the 96-well receiver plate) were prepared in HBSS buffer supplemented with 0.1% (*w/v*) bovine serum albumin (BSA; endotoxin free, Sigma-Aldrich). The plate was incubated for 1 hour at 37°C, whereupon the 96-well filter plate was removed and the neutrophils in the receiver plate were quantified using the luminescence ATP detection assay system (PerkinElmer, Waltham, MA, USA). The chemotactic index was calculated by dividing the luminescence value of the chemoattractant condition by the luminescence value of the buffer condition.

The under-agarose assay uses the migration distance of neutrophils under an agarose gel as a parameter to measure the chemotactic effect of substances (16). Agarose gels were prepared in 53 mm Ø plastic tissue culture dishes one day before the assay. To prepare the gels, equal volumes of prewarmed (48°C) solution A and solution B were mixed. Solution A contained 20% (*v/v*) FCS and 20% (*v/v*) 10x concentrated Eagle’s minimum essential medium with Earle’s salts (EMEM; Invitrogen) in pure water. Solution B consisted of 1.8% (*w/v*) agarose (Indubiose A37; Biosepra Inc., Marlborough, MA, USA) in pure water. This mixture was warmed up in a microwave until complete dissolving of the agarose. The solution was then cooled down to 48°C before mixing it with solution A. Immediately after mixing, 6 ml of solution A/B was poured per tissue culture dish; this was left for 30 minutes to cool down to RT before transfer to 4°C. The gels were left to settle overnight at 4°C. The day that the agarose assay was performed, six series (per dish) of three wells (3 mm Ø and 3 mm inter-space) were cut in the gel in a straight line using a template and a stainless steel punch with inside bevel. The agarose cores were removed with the same steel punch connected to a vacuum system. The gels were allowed to equilibrate at 37°C in a 5% CO_2_ incubator until sample dilutions and cells were prepared. Neutrophil suspension (3 × 10^7^ cells/ml) and chemokine dilutions were prepared in HBSS buffer supplemented with 1 mg/ml HSA. The center well of each series of three wells was loaded with human neutrophils (3 × 10^5^ cells in 10 µl), whereas the inner and outer wells were loaded with a buffer control and varying concentrations of chemoattractant, respectively. The agarose gels with the cells were incubated for 2 hours at 37°C/5% CO_2_, allowing sufficient time for the neutrophils to migrate towards the chemotactic gradient of the chemoattractant (distance X) or the buffer control (random migration distance Y). The migration distance (X-Y) was estimated and expressed as the percentage of maximal possible migration (3 mm).

### 2.6 Phagocytosis Assay

The capacity of purified neutrophils to phagocytose *S. aureus*-coated beads was evaluated microscopically. Black clear-bottom 96-well plates (Greiner Bio-One, Vilvoorde, Belgium) were coated with poly-L-lysine (0.1 mg/ml in sterile water; Sigma-Aldrich) for 1 hour at RT. Afterwards, the plate was washed twice with sterile distilled water and air-dried. Purified neutrophils were suspended in Live Cell Imaging solution (Invitrogen) supplemented with HEPES (20 mM) and calcein acetoxymethyl ester (Calcein AM; 1 µM; Invitrogen) to monitor viability. Subsequently, cells were added to the plate (0.05 x 10^6^ cells/well) and incubated for 30 minutes at 37°C. Afterwards, inducers (fMLF [10^-7^ M], LPS from *K. pneumoniae* [100 ng/ml] or CXCL8 (50 ng/ml) and pHrodo *S. aureus*-coated beads (62.5 µg/ml; Invitrogen) were carefully added on top of the cells. Phagocytic uptake of the beads was quantified and continuously monitored for 4 hours using the Incucyte S3 live cell imaging system v.2020A (Sartorius, Göttingen, Germany).

### 2.7 ROS Production Assay

A chemiluminescence-based assay was used to determine ROS production by neutrophils. Cells were suspended in RPMI-1640 medium without phenol red at a final concentration of 1.5 x 10^6^ cells/ml and were pre-incubated at 37°C in the presence or absence of the priming agent TNF-α (50 ng/ml). Following 10 minutes of incubation, cells were added to a white, clear-bottom, 96-well microtiter plate (PerkinElmer) and stimulated with PMA (150 ng/ml), ultrapure LPS from *E. coli* (10 µg/ml), PGN from *S. aureus* (10 µg/ml), CpG oligodeoxynucleotides (3 µM), TNF-α (50 ng/ml) or IL-1β (500 ng/ml) in the presence of 5 mM luminol (Sigma-Aldrich). Kinetic measurements of luminol oxidation were performed during 1.5 hours at 37°C using a Clariostar Monochromator Microplate Reader (BMG Labtech, Orthenberg, Germany). The results were subtracted by values obtained with PMA stimulation in the absence of luminol.

### 2.8 NETosis Assay

To assess the capacity of neutrophils to release DNA in response to stimuli, a NETosis assay was performed as described by Cockx et al. (17). In short, neutrophils were suspended at a concentration of 0.5 x 10^6^ cells/ml in RPMI-1640 medium without phenol red containing 50 nM SYTOX Green (Invitrogen). The cells were transferred to flat-bottom 96-well plates coated with poly-L-lysine (0.1 mg/ml in sterile water) and incubated for 30 minutes at 37°C to allow adherence. Subsequently, one of the following compounds was added: PMA (150 ng/ml), PGN (0.1-10 µg/ml), TNF-α (30 ng/ml), IL-1β (100 ng/ml) or IFN-γ (100 ng/ml). Additionally, for IL-1β and IFN-γ stimulation, a priming agent (TNF-α [30 ng/ml]) was included. In that case, the cells were first pre-incubated for 10 minutes at 37°C with the priming agent before adding the stimulus. After addition of the stimuli, the cells were incubated for 5 hours at 37°C and continuously monitored by the Incucyte S3 live cell imaging system. The relative area of the DNA released by neutrophils was determined using the Incucyte S3 software as described (17).

To confirm that the neutrophils released DNA due to NETosis, an additional immunofluorescence staining was performed. To this end, a 12-well tissue culture plate was filled with 18 mm Ø sterile glass coverslips. The coverslips were coated with poly-L-lysine (0.1 mg/ml in sterile water) for 1 hour at RT, washed twice with PBS and air-dried. Neutrophils were suspended in RPMI-1640 without phenol red at a concentration of 0.6 × 10^6^ cells/ml, and 900 µl was added per well. The plate was incubated for 30 minutes at 37°C to allow adherence of the cells. After 30 minutes, 100 µl of PMA (final concentration 150 ng/ml) or RPMI-1640 medium was carefully added on top of the cells. The plate was incubated for 3 hours at 37°C, whereupon the medium was removed and the cells were fixed with 4% (*w/v*) paraformaldehyde (AlfaAesar, Ward Hill, MA, USA) in HBSS buffer containing Ca^2+^ and Mg^2+^ for 15 minutes at RT. After fixation, the cells were washed twice with HBSS buffer and blocked with blocking agent (PBS + 2% [*w/v*] BSA + 10% [*v/v*] FCS) for 30 minutes at RT. The cells were then treated with 500 µl polyclonal rabbit anti-human MPO antibody (diluted 1:1000 in blocking agent; Agilent Technologies, Santa Clara, CA, USA; cat nr #A0398) and incubated overnight at 4°C in the dark. Following incubation, the cells were washed twice with HBSS buffer and incubated for 1 hour at RT with 500 µl HBSS buffer containing Hoechst (10 µg/ml; Molecular Probes, Eugene, OR, USA), Alexa Fluor 594-conjugated wheat germ agglutinin (10 µg/ml; Invitrogen) and Alexa Fluor 488-conjugated chicken-anti-rabbit antibody (dil 1:1000, Invitrogen, cat nr #A21441). Subsequently, the cells were washed three times with HBSS buffer, and the coverslips were fixed on glass microscope slides using ProLong Diamond (Invitrogen). The cells were imaged using a Zeiss fluorescence microscope with a 63x magnification objective.

### 2.9 Neutrophil Degranulation and CXCL8 Production Assays

Neutrophils were induced with a variety of stimuli to assess their capacity to release granular proteins and to produce cytokines. Neutrophils were suspended at a concentration of 1 × 10^6^ cells/ml in RPMI-1640 medium containing 10% (*v/v*) FCS, GM-CSF (5 ng/ml) and one of the following compounds: PGN (1 µg/ml), LPS from *E. coli* (5 µg/ml), IL-1β (10 ng/ml), TNF-α (10 ng/ml) or IFN-γ (10 ng/ml). After 2 and 24 hours, the cells were collected and spun down (5 min, 16000g). The supernatants were stored at -20°C until further analysis.

Release of NE and MPO after 2 hours of stimulation was determined by commercially available DuoSet ELISAs (R&D, Minneapolis, MN, USA; resp. cat nr DY9167 and DY3174), according to the manufacturer’s instructions. The concentration of CXCL8 in the supernatant after 24 hours of stimulation was measured by a sandwich ELISA developed in-house (18).

### 2.10 Phenotypical Analysis of Neutrophils

2 × 10^5^ purified neutrophils were incubated with 1:20 diluted FcR block (Miltenyi Biotec, Bergisch Gladbach, Germany) and 1:1000 diluted Zombie Aqua 516 (Biolegend, San Diego, CA, USA) or 1:20000 diluted Fixable Viability Stain 620 (BD Biosciences) in PBS for 15 minutes at RT. Subsequently, cells were washed twice with flow cytometry buffer (PBS + 2% [*v/v*] FCS + 2 mM EDTA) and stained with fluorescently labeled antibodies. Antibodies used in this study were titrated in-house and are listed in **Supplementary Table 1**. Following incubation for 25 minutes at 4°C, cells were washed twice with flow cytometry buffer and fixed with BD Cytofix (BD Biosciences). Samples were run on a BD LSRFortessa™ X-20 equipped with DIVA software. FlowJo software was used for downstream analysis. Neutrophils were gated as CD16^+^CD66b^+^ cells within the population of live, single cells (**Supplementary Figure 1**).

### 2.11 Statistics

No normal distribution of data was detected as evaluated by Shapiro-Wilk tests. Wilcoxon matched-pairs signed rank tests were used to determine statistical differences between neutrophils purified with immunomagnetic or density-gradient separation. Statistical tests for comparison were two-sided, and p < 0.05 was considered significant. Data are shown as box-and-whiskers plots with the central line in the box representing the median, while the box spands the interquartile range, with the whiskers indicating the full distribution of the data. Alternatively, results from each individual donor are connected by lines. All outliers were included in the data and all data points are shown. Statistical analysis and visualization of the data was performed using GraphPad Prism 9.3.1.

### 2.12 Study Approval

The study was approved by the Ethics Committee of the University Hospital Leuven, and all participants signed an informed consent form according to the ethical guidelines of the Declaration of Helsinki (study number: S58418).

## 3 RESULTS

### 3.1 Neutrophils Purified by Immunomagnetic Isolation Are More Responsive to Chemoattractants in Polarization Assays but Not in Migration Assays

**Figure 1:**
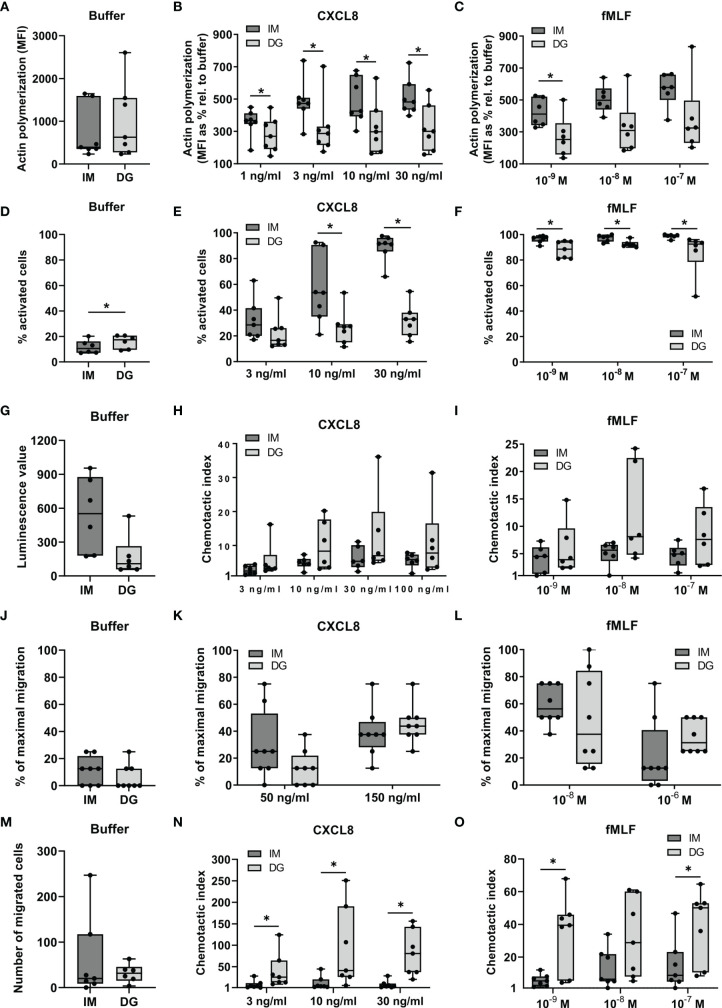
Neutrophils purified by immunomagnetic isolation are more responsive to chemoattractants in polarization assays but not in migration assays. Peripheral blood neutrophils were isolated by immunomagnetic (IM) or density-gradient (DG) purification. **(A–C)** A flow cytometric assay was used to quantify the content of polymerized (F-)actin in the cells. Neutrophils in suspension were stimulated with CXCL8, fMLF or buffer (pre-warmed RPMI-1640 medium supplemented with 1 mg/ml HSA) for 30 seconds. Fixed and permeabilized cells were stained with Alexa Fluor 555 Phalloidin, which selectively binds to polymerized actin. **(A)** Results from unstimulated neutrophils (only buffer) are represented as median fluorescence intensities (MFI). **(B, C)** Results from chemoattractant-stimulated neutrophils are represented as the MFIs relative to buffer control (in %). **(D–F)** Shape change assays were used to assess the morphological changes occurring in neutrophils in response to chemoattractants. Neutrophils in suspension were **(D)** left untreated or stimulated with **(E)** CXCL8 or **(F)** fMLF for a period of 3 minutes. Results are represented as the % activated neutrophils. **(G–O)** The **(G, J, M)** spontaneous and **(H–I, K–L, N–O)** chemoattractant-induced migratory response of neutrophils was evaluated in three different *in vitro* chemotaxis assays, namely: **(G–I)** the Multiscreen chemotaxis assay, **(J–L)** the under-agarose assay and **(M–O)** the 48-well Boyden chamber chemotaxis assay. Results are represented as **(G)** the luminescence value, **(J–L)** % of maximal migration, **(M)** number of migrated cells or **(H, I, N, O)** chemotactic indices (CI). Data are shown as box-and-whisker plots (box: median with interquartile range, whiskers: full data distribution), with each dot representing an individual healthy donor (n = 6-8). Results were statistically analyzed by Wilcoxon matched-pairs signed rank test (*p < 0.05 for statistical differences between neutrophils purified with immunomagnetic or density-gradient separation).

To compare the chemoattractant sensitivity of neutrophils purified through density-gradient centrifugation *versus* immunomagnetic separation, we performed two different polarization assays: actin polymerization and shape change assays. The former quantifies the cellular content of polymerized (F-)actin while the latter detects visible morphological changes in neutrophils as a measure of activation. Baseline F-actin contents did not significantly differ between neutrophils purified by density-gradient centrifugation or immunomagnetic isolation (**Figure 1A**). Stimulation by CXCL8 or fMLF increased the levels of F-actin in neutrophils purified by both methods, but this increase was significantly greater in immunomagnetically purified cells (**Figures 1B, C**). Compared to density-gradient-derived cells, neutrophils isolated through immunomagnetic separation showed less pronounced morphological signs of activation at baseline, but were significantly more responsive to CXCL8 (10-30 ng/ml) and fMLF (10^-9^–10^-7^ M) in shape change assays (**Figures 1D**–**F**).

Next, we compared the migratory properties of isolated neutrophils in three different *in vitro* chemotaxis assays: the 96-well Multiscreen assay, the under-agarose assay and the 48-well Boyden assay (**Figures 1G**–**O**). The Multiscreen and the Boyden assay are both based on vertical migration of neutrophils through a membrane. The substantial difference between these approaches is that the Boyden assay only allows to microscopically count cells adhered to the lower side of the membrane. The Multiscreen technique, on the other hand, uses chemiluminescence to quantify all cells that have passed the membrane to the lower compartment in response to chemoattractants. The under-agarose assay examines horizontal migration of the neutrophils along a chemoattractant gradient, the extent of which is estimated through microscopical examination.

The spontaneous migratory responses in all three assays were not significantly different between neutrophils derived from immunomagnetic and density-gradient separation (**Figures 1G, J, M**). The migratory response towards CXCL8 or fMLF was not significantly different between neutrophils purified by immunomagnetic and density-gradient separation in the 96-well Multiscreen and the under-agarose assay (**Figures 1H, I, K, L**). However, neutrophils purified by immunomagnetic beads showed a significantly reduced potency to migrate towards CXCL8 and fMLF in the Boyden chamber chemotaxis assay (**Figures 1N, O**).

### 3.2 Neutrophils Purified by Immunomagnetic Isolation Show Increased Phagocytosis of Bacteria-Coated Beads

**Figure 2:**
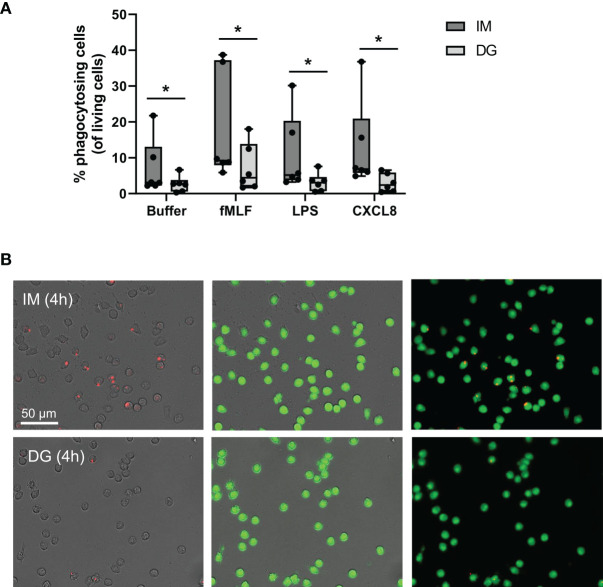
Neutrophils purified by immunomagnetic isolation show increased phagocytosis of bacteria-coated beads. Peripheral blood neutrophils were isolated by immunomagnetic (IM) or density-gradient (DG) purification. The capacity of neutrophils to actively engulf extracellular particles was quantified using *S. aureus*-coated beads and imaged using the Incucyte S3 live cell imaging system. **(A)** Neutrophils were additionally stimulated with buffer, fMLF (10^-7^ M), LPS (100 ng/ml) or CXCL8 (50 ng/ml). **(B)** Representative image of phagocytosed beads (red) and purified, living neutrophils (green) after 4 hours of stimulation with fMLF. Results are represented as the percentage phagocytosing neutrophils normalized to the amount of living cells. Data are shown as box-and-whisker plots (box: median with interquartile range, whiskers: full data distribution), with each dot representing an individual healthy donor (n = 6) and were statistically analyzed by Wilcoxon matched-pairs signed rank test (*p < 0.05 for statistical differences between neutrophils purified with immunomagnetic or density gradient separation).

Purified neutrophils were stimulated with the chemokine CXCL8 or bacterial products (LPS, fMLF), and the phagocytosis of *S. aureus*-coated beads was monitored for 4 hours (**Figures 2A, B**). Spontaneous uptake of beads, displayed as buffer condition, was significantly higher in neutrophils purified with immunomagnetic separation compared to density-gradient-derived cells. Furthermore, after stimulation with fMLF, LPS or CXCL8, the increased phagocytosis of beads by cells purified by immunomagnetic selection is maintained (**Figures 2A, B**).

### 3.3 Neutrophils Purified by Immunomagnetic Isolation Release More ROS Following Priming With TNF-α

**Figure 3:**
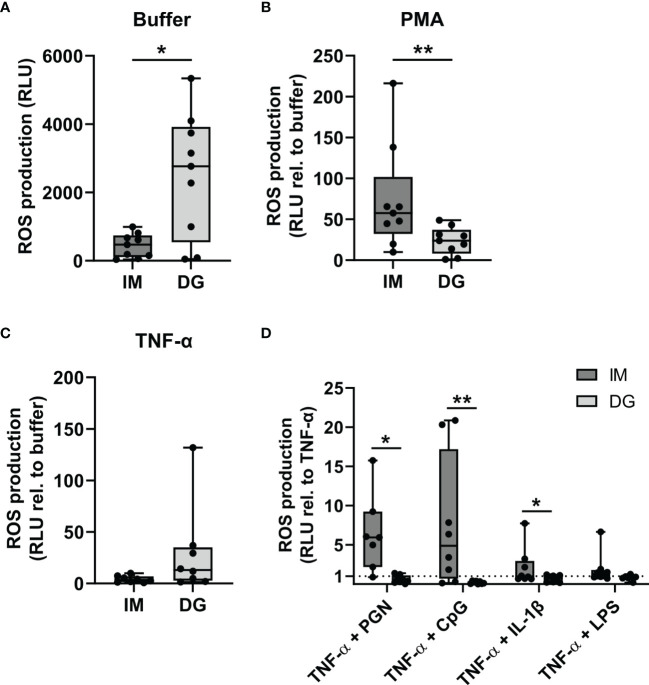
Neutrophils purified by immunomagnetic isolation release more ROS following priming with TNF-α. Peripheral blood neutrophils were isolated by immunomagnetic (IM) or density-gradient (DG) purification. ROS production was assessed by kinetic measurements of luminol oxidation during 1.5 hours after stimulation with **(A)** buffer, **(B)** 150 ng/ml PMA or **(C)** 50 ng/ml TNF-α. Results are represented as relative light units (RLU) for unstimulated neutrophils (only buffer) or as the fold change in RLU relative to the corresponding buffer values for the stimulated neutrophils. **(D)** Neutrophils were pretreated (primed) with 50 ng/ml TNF-α during 10 minutes at 37°C, followed by stimulation with 10 µg/ml PGN, 3 µM CpG, 500 ng/ml IL-1β, or 10 µg/ml LPS. Kinetic measurements of luminol oxidation were performed during 1.5 hours. Results are represented as the fold change in RLU relative to stimulation with TNF-α alone. Data are shown as box-and-whisker plots (box: median with interquartile range, whiskers: full data distribution), with each dot representing an individual healthy donor (n = 8-9). Results were statistically analyzed by Wilcoxon matched-pairs signed rank test (*p < 0.05; **p < 0.01 for statistical differences between neutrophils purified with immunomagnetic or density-gradient separation).

To evaluate ROS generation by purified neutrophils, a chemiluminescence-based assay was employed. Density-gradient-derived neutrophils showed enhanced basal ROS production compared to cells isolated by immunomagnetic separation (**Figure 3A**). Upon PMA stimulation, neutrophils from both purifications readily produced ROS, but the production was significantly higher by cells derived from immunomagnetic purification (**Figure 3B**). Priming with TNF-α caused a 5-10 fold increase in ROS production compared to the buffer condition independently of the purification method (**Figure 3C**). Next, we examined the capacity of TNF-α-primed neutrophils to produce ROS in response to subsequent stimulation with cytokines (IL-1β) or pathogen-associated molecular patterns (CpG, LPS, and PGN). Immunomagnetic isolation-derived neutrophils primed with TNF-α produced substantially higher ROS levels upon stimulation with PGN or CpG in most of the donors (**Figure 3D**). Only a moderate increase in ROS production was seen upon IL-1β or LPS stimulation of immunomagnetic isolation-derived neutrophils primed with TNF-α. In stark contrast to neutrophils isolated by immunomagnetic purification, TNF-α-primed density-gradient-derived neutrophils failed to enhance ROS production upon subsequent stimulation with IL-1β, CpG, LPS, or PGN (**Figure 3D**).

### 3.4 Neutrophils Purified by Immunomagnetic Isolation Release Stimulus-Induced NETs More Readily

**Figure 4:**
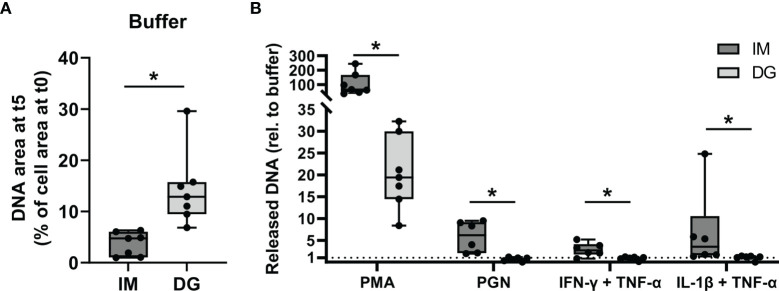
Neutrophils purified by immunomagnetic isolation release stimulus-induced NETs more readily. Peripheral blood neutrophils were isolated by immunomagnetic (IM) or density-gradient (DG) purification. The cells were unprimed (buffer, PMA, PGN conditions) or primed with TNF-α (30 ng/ml) for 10 minutes (IFN-γ, IL-1β conditions). Subsequently, the neutrophils were exposed for 5 hours to **(A)** buffer or **(B)** PMA (150 ng/ml), PGN (10 µg/ml), IFN-γ (100 ng/ml) or IL-1β (100 ng/ml) and the release of NETs was monitored using the Incucyte S3 live cell imaging system. **(A, B)** The area of the released DNA after 5 hours was measured and corrected for the amount of DNA present at the start of the exposure. Data for the stimuli are presented as fold change relative to the corresponding buffer values. Data are shown as box-and-whisker plots (box: median with interquartile range, whiskers: full data distribution), with each dot representing an individual healthy donor (n = 7). Results were statistically analyzed by Wilcoxon matched-pairs signed rank test (*p < 0.05 for statistical differences between neutrophils purified with immunomagnetic or density-gradient separation).

To compare the capacity of purified neutrophils to release NETs, neutrophils were exposed to cytokines (TNF-α, IL-1β, IFN-γ) or bacterial components (PGN) for a period of 5 hours, during which the release of DNA was monitored. By staining extracellular DNA, NETs could efficiently be detected, as confirmed by immunofluorescent co-staining of DNA and MPO (**Supplementary Figure 2**). PMA, a direct activator of protein kinase C (PKC) was used as a positive control. Upon buffer stimulation, significantly less free DNA was released by immunomagnetic beads-purified neutrophils as compared to density-gradient-derived neutrophils (**Figure 4A**). Upon stimulation with PMA, neutrophils purified with either method underwent NETosis, although the amount of extracellular DNA was significantly higher in immunomagnetic beads-purified neutrophils as compared to density-gradient-derived neutrophils (**Figure 4B**). PGN (10 µg/ml), IL-1β and IFN-γ (the latter two in combination with TNF-α priming) induced NET formation in immunomagnetic bead-purified neutrophils but not in density-gradient-derived neutrophils (**Figure 4B**). Lower concentrations of PGN (0.1-1 µg/ml) or TNF-α, IFN-γ and IL-1β alone did not result in NET formation by neutrophils isolated with either method (data not shown).

### 3.5 Neutrophils Purified by Immunomagnetic Isolation Show Less Spontaneous But More Stimulus-Provoked Degranulation

**Figure 5:**
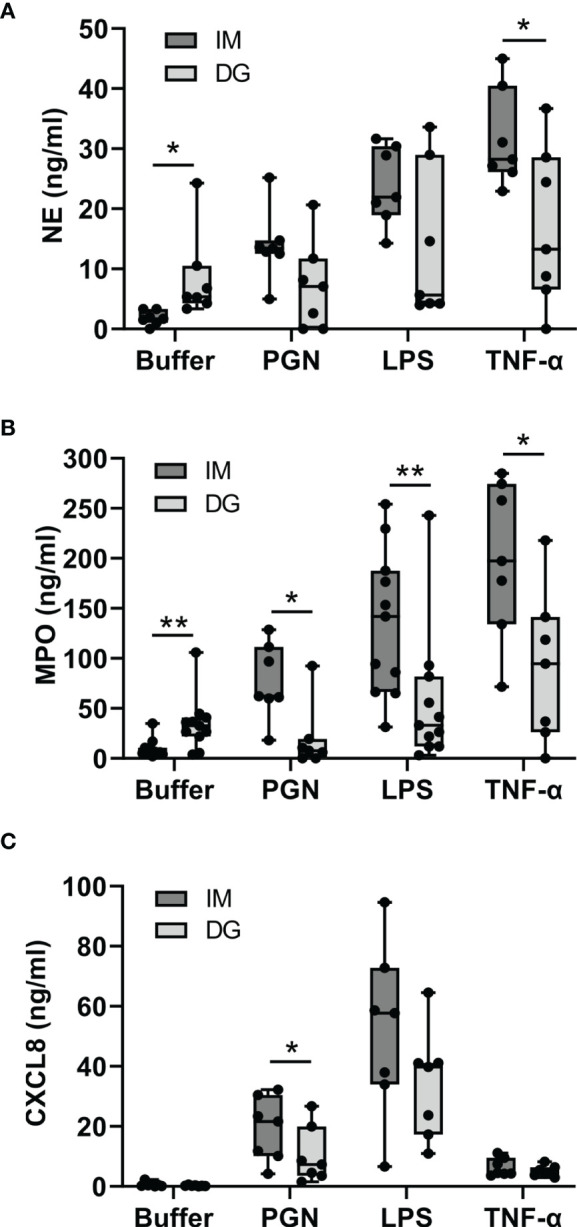
Neutrophils purified by immunomagnetic isolation show less spontaneous but more stimulus-provoked degranulation, whereas CXCL8 is produced irrespective of the purification method. Peripheral blood neutrophils were isolated by immunomagnetic (IM) or density-gradient (DG) purification and stimulated for **(A, B)** 2 hours or **(C)** 24 hours with buffer, PGN (1 µg/ml), LPS (5 µg/ml) or TNF-α (10 ng/ml). Subsequently, the concentration of **(A)** neutrophil elastase (NE), **(B)** myeloperoxidase (MPO) and **(C)** CXCL8 in the cell culture supernatant was determined by ELISA. Data are shown as box-and-whisker plots (box: median with interquartile range, whiskers: full data distribution), with each dot representing an individual healthy donor (n = 7-11). Statistical differences were determined by Wilcoxon matched-pairs signed rank test (*p < 0.05; **p < 0.01 for statistical differences between neutrophils purified with immunomagnetic or density gradient separation).

Neutrophils were stimulated with cytokines (TNF-α, IL-1β, IFN-γ) or Toll-like receptor (TLR) ligands (LPS, PGN) for 2 hours, whereupon the levels of NE and MPO were measured in the supernatant (**Figures 5A, B**). NE and MPO are stored in azurophilic granules and are released through degranulation (19). Compared to cells purified with immunomagnetic beads, density-gradient-derived neutrophils spontaneously released significantly higher amounts of NE and MPO. In contrast, when stimulated with pro-inflammatory stimuli, immunomagnetic beads-purified neutrophils released significantly more MPO (in response to PGN, LPS and TNF-α) and NE (in response to TNF-α). IL-1β and IFN-γ did not evoke any degranulation responses in neutrophils isolated with either method in our setup (data not shown).

### 3.6 Neutrophils Produce CXCL8 in Response to Cytokines and TLR Ligands Irrespective of the Purification Method

Neutrophils were stimulated with cytokines (TNF-α, IL-1β, IFN-γ) or TLR ligands (LPS, PGN) for 24 hours, whereupon the levels of CXCL8 in the supernatant were measured. CXCL8 is normally not contained in neutrophil granules but must be synthesized *de novo* in order to be released (20). Unstimulated neutrophils purified with immunomagnetic or density-gradient methods produced virtually no CXCL8. In contrast, stimulation with LPS, PGN or TNF-α evoked substantial CXCL8 production. The levels of CXCL8 tended to be higher in neutrophils purified with immunomagnetic beads (**Figure 5C**). IL-1β and IFN-γ did not trigger the production of CXCL8 in our setup (data not shown).

### 3.7 Neutrophils Acquire an Activated Phenotype and Partially Express Atypical Markers After Density-Gradient Purification

**Figure 6:**
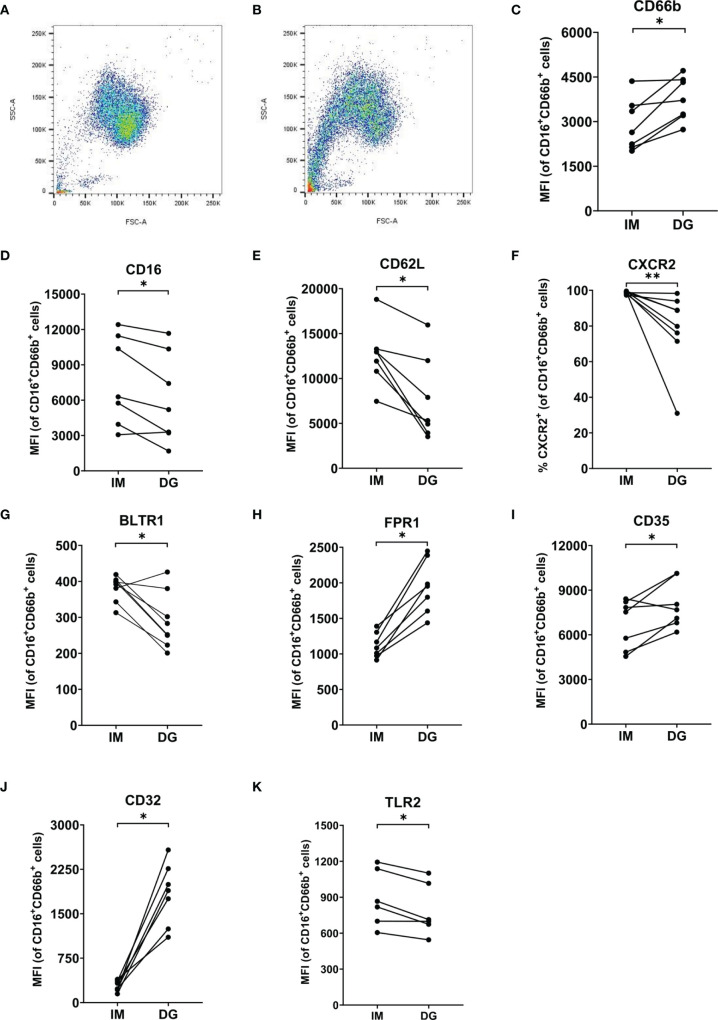
Neutrophils purified by density-gradient centrifugation display an activated phenotype. Flow cytometry was used to evaluate the **(A, B)** size (FSC), granularity (SSC) and surface expression of **(C)** CD66b, **(D)** CD16, **(E)** CD62L, **(F)** CXCR2, **(G)** BLTR1, **(H)** FPR1, **(I)** CD35, **(J)** CD32 and **(K)** TLR2 on neutrophils (gated as CD16^+^CD66b^+^ cells) isolated by immunomagnetic (IM) or density-gradient purification (DG) from peripheral blood of healthy donors. Results are represented as percentage of neutrophils positive for the marker or median fluorescence intensity (MFI). Results from each individual donor (n = 6-8) are connected by lines and were statistically analyzed by Wilcoxon matched-pairs signed rank test (*p < 0.05; **p < 0.01 for statistical differences between neutrophils purified with immunomagnetic or density-gradient separation).

Purified peripheral blood neutrophils (defined as live CD16^+^CD66b^+^ cells) were phenotypically characterized using multicolor flow cytometry, with a focus on the expression of adhesion molecules, activation/maturation markers and chemoattractant receptors. As expected, >90% of neutrophils from healthy donors were mature cells (CD10^+^) (**Supplementary Figure 3A**). However, purification method-dependent changes in neutrophil phenotype were observed. Density-gradient purification, but not immunomagnetic isolation, induced a partial shift towards reduced size (lower forward scatter [FSC]) and granularity (lower side scatter [SSC]) (**Figures 6A, B**). In addition, neutrophils derived from density-gradient purification expressed a distinct expression pattern of multiple markers related to activation (**Figures 6C**–**J**). The most prominent characteristics of density-gradient-derived neutrophils include increased expression of CD66b (a granulocyte-specific surface molecule), decreased levels of CD16 (a low-affinity Fcγ receptor) and partial downregulation of CD62L (L-selectin) (**Figures 6C**–**E**). This expression pattern points to activation of neutrophils, as reported in literature (19). No significant differences were found in the expression of the integrin chain CD11b between neutrophils isolated by immunomagnetic and density-gradient purification (**Supplementary Figure 3B**). A remarkable difference between neutrophils derived from immunomagnetic and density-gradient isolation was the altered expression of chemoattractant receptors. Density-gradient purification (but not immunomagnetic isolation purification) yielded a large proportion of cells that completely lacked the chemokine receptor CXCR2 (**Figure 6F**). This was in contrast to chemokine receptor CXCR1, which was present on all cells derived from both purification methods (**Supplementary Figure 3C**). Both CXCR1 and CXCR2 surface expression levels per cell seemed to be slightly lower on density-gradient-derived cells, but these data did not reach significance (**Supplementary Figures 3D, E**). Furthermore, density-gradient-derived neutrophils were characterized by decreased leukotriene B4 receptor (BLTR1), increased formyl peptide receptor 1 (FPR1) and a trend towards lower expression of complement receptor C5aR, as compared to immunomagnetic isolation-derived cells (**Figures 6G**–**H** and **Supplementary Figure 3F**). Other markers that were more abundantly expressed on density-gradient-derived cells than on those purified by immunomagnetic isolation included complement receptor 1 (CD35) and the Fcγ receptor CD32 (**Figures 6I, J**). No significant differences were found in the expression of Sialyl-Lewis X (CD15), the tetraspanin CD63 and the Fcγ receptor CD64 (**Supplementary Figures 3G**–**I**). Finally, we measured the expression levels of several TLRs on neutrophils. Compared to cells purified by immunomagnetic isolation, density-gradient-derived neutrophils expressed lower levels of TLR2, but similar levels of TLR4, TLR6 and TLR9 (**Figure 6K** and **Supplementary Figures 3J**–**L**).

**Figure 7:**
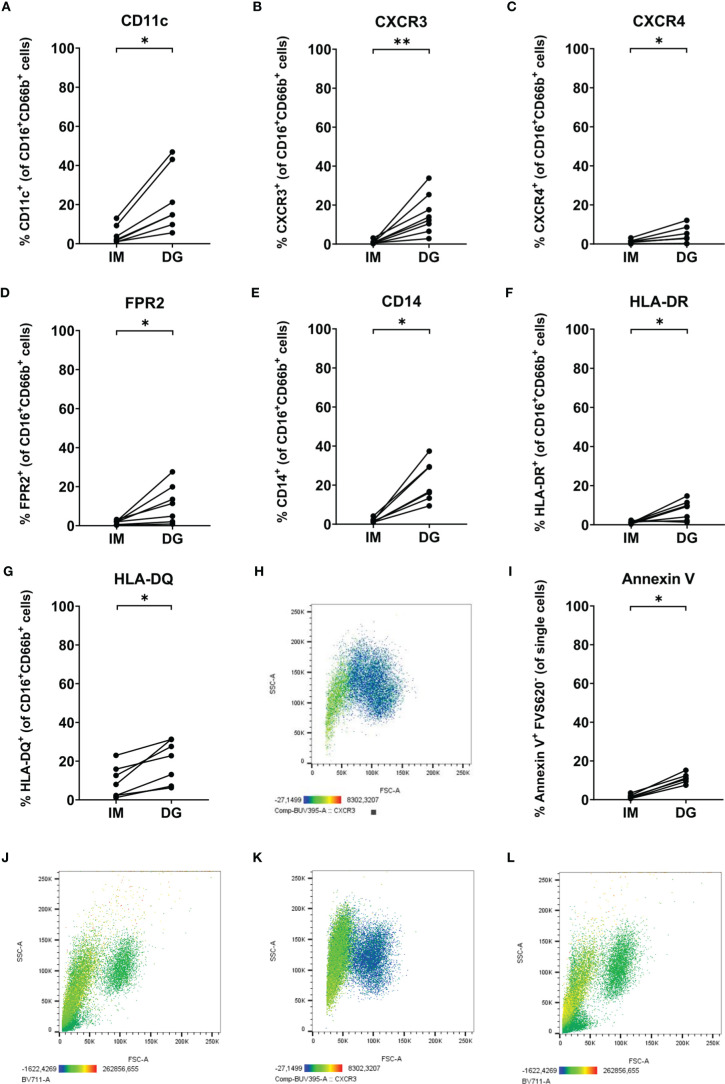
Neutrophils purified by density-gradient centrifugation partially express markers atypical for neutrophils. Flow cytometry was used to evaluate the surface expression of **(A)** CD11c, **(B)** CXCR3, **(C)** CXCR4, **(D)** FPR2, **(E)** CD14, **(F)** HLA-DR and **(G)** HLA-DQ on neutrophils (gated as CD16^+^CD66b^+^ cells) isolated by immunomagnetic (IM) or density-gradient purification (DG) from peripheral blood of healthy donors. **(H)** CXCR3 (BUV395) staining (indicated by color code below the plot) on FSC/SSC plot of neutrophils purified by density-gradient centrifugation. **(I)** Annexin V and FVS620 staining on neutrophils isolated by IM or DG purification from peripheral blood of healthy donors. **(J)** Annexin V (BV711) staining (indicated by color code below the plot) on FSC/SSC plot of neutrophils purified by density-gradient centrifugation. **(K, L)** CXCR3 (BUV395) and Annexin V (BV711) staining (indicated by color code below the plot) on FSC/SSC plot of neutrophils purified by immunomagnetic purification immediately after a density-gradient purification. Results are represented as percentage of neutrophils positive for the marker. Results from each individual donor (n = 6-8) are connected by lines and were statistically analyzed by Wilcoxon matched-pairs signed rank test (*p < 0.05; **p < 0.01 for statistical differences between neutrophils purified with immunomagnetic or density-gradient separation).

Remarkably, density-gradient-derived neutrophils expressed several markers that are generally absent on neutrophils purified with the immunomagnetic method (**Figure 7**). We detected significant upregulation of CD11c (an integrin chain), chemokine receptors CXCR3 and CXCR4, FPR2, CD14 (a co-receptor for TLR4), and the antigen-presenting molecules HLA-DR and HLA-DQ (**Figures 7A**–**G**). It is worth mentioning that these atypical markers were mainly present on the smaller and less granular neutrophil population (20-45% of all neutrophils) that was only detected in cells purified by density-gradient centrifugation (**Figure 7H** and **Supplementary Figures 4**, **5**). Moreover, compared to cells isolated using immunomagnetic beads, density-gradient-derived neutrophils contained a significantly higher proportion of cells that were positive for Annexin V but negative for the live/dead marker FVS620 (**Figure 7I**). These cells also represented a part of the small neutrophil population expressing neutrophil atypical markers (**Figure 7J** and **Supplementary Figure 4I**).

Next, we wanted to exclude the possibility that the observed phenotypic differences between neutrophils purified by the two different methods merely resulted from removal of cells with atypical markers during the immunomagnetic isolation. For example, CD14 is a marker which is commonly found on monocytes. Therefore, it could be possible that the immunomagnetic bead cocktail contains antibodies against CD14, removing not only the contaminating monocyte population but also CD14-expressing neutrophils – if present. To test this hypothesis, we performed a second, immunomagnetic, purification step on neutrophils isolated by density-gradient centrifugation. Following the additional purification step, we still observed the presence of the low-FSC/SSC neutrophil population expressing atypical surface markers (**Figures 7K, L**). Hence, we can conclude that the atypical marker expression is likely induced by the density-gradient purification method rather than being masked by the immunomagnetic bead isolation.

## 4 Discussion

Neutrophils, representing an indispensable part of the mammalian immune system, can eliminate pathogens through phagocytosis, production of ROS, release of NETs, and secretion of antibacterial peptides and enzymes (2). The short life span of neutrophils and their high sensitivity to external stimuli pose a challenge to those aiming to study these cells *in vitro* (8). However, an extensive comparison of the functionality of neutrophils purified by different techniques has so far not been performed.

In this study, we compared the functional characteristics of neutrophils purified by two routinely used purification methods: (I) density-gradient centrifugation followed by dextran sedimentation and hypotonic lysis of erythrocytes; and (II) immunomagnetic isolation based on negative selection. We found that immunomagnetic isolation-derived unstimulated neutrophils exhibited reduced baseline polarization and spontaneously released less ROS, NETs and granular proteins as compared to unstimulated density-gradient-derived cells. When stimulated with pro-inflammatory mediators, immunomagnetic beads-isolated neutrophils polarized more readily and released more ROS, NETs and granular proteins than stimulated density-gradient-derived cells. In addition, immunomagnetically purified neutrophils showed enhanced phagocytosis of *S. aureus-*coated beads. No major differences in CXCL8 production were found between neutrophils isolated by the two methods. A summary of the results can be found in **Table 1**. Overall, neutrophils separated using immunomagnetic beads seemed to be less activated at baseline but more responsive to activating stimuli than density-gradient-derived neutrophils. This was further supported by the observation that density-gradient-derived neutrophils displayed higher levels of activation markers (*e.g.* CD66b, FPR1, CD35, CD11c) on their surface. Together, these results suggest that neutrophils purified by immunomagnetic separation most closely resemble the quiescent cells found in the bloodstream and are therefore more representative of naïve blood neutrophils.

**Table 1:** Summary of the results.

Neutrophil function	Spontaneous	In response to stimulus
Cell polarization	IM < DG	IM > DG
Migration	IM ≈ DG	IM ≈ DG
Phagocytosis	/	IM > DG
ROS production	IM < DG	IM > DG
NETosis	IM < DG	IM > DG
Degranulation	IM < DG	IM > DG
Cytokine production	IM ≈ DG	IM ≈ DG

Neutrophils purified by immunomagnetic isolation (IM) and density-gradient centrifugation (DG) were subjected to several functional tests. The baseline activation in the test (spontaneous) and stimulus-induced activation (in response to stimulus) were compared. IM < DG means the response was lower in IM-derived cells than in DG-derived cells; IM > DG means the response was higher in IM-derived cells than in DG-derived cells; / means the response was not assessed; IM ≈ DG means the response was comparable in IM-derived and DG-derived neutrophils.

Having found density-gradient-derived neutrophils to be artificially activated, we further explored the expression of typical and atypical surface markers on neutrophils. Density-gradient-derived neutrophils expressed lower levels of BLTR1, CD62L, CXCR2, CD16 and TLR2 and higher levels of CXCR4, FPR1, CD11c, CD66b, CD32 and CD35. The lower expression levels of CXCR2 and TLR2 on density gradient-derived neutrophils could partially explain the reduced response to CXCL8 and PGN in the different functional assays. However, this is not the case for FPR1, which was more highly expressed on density-gradient-derived cells, despite most functional responses to fMLF being decreased as compared to immunomagnetically purified neutrophils. Nevertheless, increased receptor expression does not necessarily exclude reduced responsiveness. Indeed, enhanced levels of CCR1 and/or CCR5 were shown to be present on monocytes from hemolytic uremic syndrome (HUS) or chronic obstructive pulmonary disease (COPD) patients, even though HUS or COPD monocytes did not respond as well as monocytes from healthy individuals in functional assays (21, 22).

In addition, we found that a significant proportion of neutrophils (up to 45% of all neutrophils, as defined by expression of both CD16 and CD66b), present among density-gradient-derived cells but not immunomagnetically purified cells, expressed a range of atypical receptors, including CXCR3, FPR2, CD14 and MHC class II molecules. Using a two-step purification procedure, we showed that the expression of these markers was specifically induced by density-gradient purification, and not masked by immunomagnetic bead purification. The atypical markers in question are involved in many different diseases (23–26). Thus, if their expression on healthy peripheral blood neutrophils is induced by the density-gradient isolation method, this should be kept in mind to avoid bias when studying patient samples. Cells expressing these atypical markers were typically smaller in size and less granular compared to the rest of the neutrophil population. To assess whether this atypical marker expression could be explained by neutrophil apoptosis, we performed an additional staining with annexin V and found a small percentage (5-15%) of density-gradient-derived neutrophils to be annexin V-positive. We believe that this small population of neutrophils comprises aging (CXCR4^+^) neutrophils which are slowly going into apoptosis (27). Of note, we did not include a positive control for the annexin V staining and therefore have no indication of the signal intensity we measured. For mast cells, it is known that a high degree of degranulation can also lead to the appearance of phosphatidylserine on the outer cell membrane and as such annexin V binding (28). Whether a similar phenomenon occurs in neutrophils is as of yet unknown; therefore our findings should be interpreted with care.

Despite significant differences in stimulus-induced polarization, we found no difference in Multiscreen and under-agarose migration of neutrophils isolated by the two different purification methods. These findings point to other mechanisms that may compensate for the lack of polarization in density-gradient-derived neutrophils in the process of migration. Surprisingly, not all three chemotaxis assays showed the same results. While immunomagnetically and density-gradient-derived neutrophils migrated equally well in the Multiscreen and under-agarose assay, relatively little chemotaxis by immunomagnetically purified neutrophils was observed in the Boyden chamber assay. This observation could not be attributed to lower levels of the chemokine receptors CXCR1 or CXCR2, both receptors for CXCL8. The increased chemotactic migration of density-gradient-derived neutrophils towards fMLF could potentially be explained by the increased expression of FPR1 on the membrane compared to immunomagnetically purified neutrophils, but this is not reflected in the actin polymerization and shape change assays. However, it should be noted that very few neutrophils purified with immunomagnetic beads were visible on the Boyden microchamber membrane after staining. Therefore, it is possible that these neutrophils did migrate but did not remain attached to the membrane due to different expression levels of adhesion molecules. This hypothesis is supported by the observation that density-gradient-derived neutrophils expressed higher levels of the integrin CD11c, and by the fact that activated neutrophils tend to express integrins in an open conformation, facilitating adhesion (29).

The profound differences in functionality and receptor expression of neutrophils purified by immunomagnetic and density-gradient methods are important, as literature reports describe neutrophils in the circulation to be largely quiescent (7). Therefore, neutrophils purified by immunomagnetic beads may be a more accurate representation of peripheral blood neutrophils *in vivo*. However, the exact mechanism causing the activation of neutrophils purified by density-gradient centrifugation remains as of yet unclear. An obvious difference between both purification methods is the time required for the purification: *circa* 1 hour for immunomagnetic separation *versus circa* 3 hours in the case of the density-gradient method. However, time alone cannot be responsible for the difference in activation, as immunomagnetically purified neutrophils left at room temperature for 2 hours after the purification did not show upregulation of activation markers to a similar extent as was seen in density-gradient-derived neutrophils (data not shown).

Other factors implicated in density-gradient purification only are Pancoll, dextran, repeated centrifugation steps and hypotonic lysis of erythrocytes. Previous research showed that dextran sedimentation may cause neutrophil activation if it is performed prior to density-gradient centrifugation, since monocytes from blood can become activated and release TNF-α, which in turn stimulates the neutrophils (30). Whether contaminating eosinophils or basophils (which are isolated by the density-gradient purification method together with the neutrophils) exhibit a similar neutrophil-activating effect, remains yet to be investigated. Another important difference between both purification methods is that immunomagnetic bead separation does not lead to the loss of low-density neutrophils (LDNs), which normally end up in the PBMC layer and are removed during density-gradient separation of neutrophils. Immune suppression (in particular suppression of T cells) is one of the functions ascribed to LDNs (31). We should also mention that the percentage of LDNs in healthy individuals is typically less than 3% (32) and is therefore unlikely to affect neutrophil function to the extent of our findings. Another study found that free MPO can activate neutrophils by binding to the CD11b/CD18 integrin (33). MPO is stored in the azurophilic granules and is released through degranulation upon stimulation of the neutrophil (34). Repeated centrifugation steps of neutrophils during density-gradient isolation may lead to physical disruption of some cells and artificial release of MPO. This process could initiate a positive feedback loop, activating other neutrophils and triggering premature ROS release and NETosis. This theory could explain the spontaneously increased release of NETs, ROS and granular proteins of unstimulated density-gradient-derived neutrophils and the low response of stimulated density-gradient-derived neutrophils in most functional assays, compared to immunomagnetic beads-derived neutrophils. We anticipate that the initial MPO could have been released by the less granular cell population present among density-gradient-derived neutrophils. In addition to the mechanical stress due to repeated centrifugation, release of damage-associated molecular patterns by lysed red blood cells may stimulate neutrophils during density-gradient purification (35, 36).

In conclusion, we have compared the effects of immunomagnetic and density-gradient purification on neutrophil phenotype and functionality. The two purification methods had similar yield and purity of neutrophils. We found that peripheral blood neutrophils isolated by immunomagnetic purification were generally less activated as compared to those isolated by density-gradient purification, and responded more readily to activating stimuli in functional assays. Based on our results, we recommend using the faster immunomagnetic separation with negative selection to test neutrophil polarization, phagocytosis, ROS production, degranulation response and NETosis. For Boyden chamber migration assays, we recommend using density-gradient centrifugation. For Multiscreen chemotaxis, under-agarose and cytokine production assays both neutrophil purification methods are suitable. Our study highlights the importance of choosing the correct methodology for the unbiased study of neutrophils *in vitro*.

## Data Availability Statement

The original contributions presented in the study are included in the article/**Supplementary Material**. Further inquiries can be directed to the corresponding author.

## Ethics Statement

The studies involving human participants were reviewed and approved by Ethics Committee of the University Hospital Leuven. The patients/participants provided their written informed consent to participate in this study.

## Author Contributions

Experiment design: PM, SS, PP, and MG. Execution of experiments and data analysis: MB, SC, MDB, LV, NP, SAS, MM, and MG. Manuscript writing: MB, SC, MDB, SAS, MM, and MG. Writing – review & editing: all authors. All authors approved the submitted version.

## Funding

This research was supported by a C1 grant of KU Leuven (C16/17/010) and a research project of FWO-Vlaanderen (G080818N). MB, SC, and MDB are supported by PhD fellowships and SAS by a postdoctoral fellowship of FWO-Vlaanderen. MM is supported by a L’Oréal/UNESCO/FWO PhD fellowship For Women in Science. The funder was not involved in the study design, collection, analysis, interpretation of data, the writing of this article or the decision to submit it for publication.

## Conflict of Interest

The authors declare that the research was conducted in the absence of any commercial or financial relationships that could be construed as a potential conflict of interest.

## Publisher’s Note

All claims expressed in this article are solely those of the authors and do not necessarily represent those of their affiliated organizations, or those of the publisher, the editors and the reviewers. Any product that may be evaluated in this article, or claim that may be made by its manufacturer, is not guaranteed or endorsed by the publisher.
