# Supplementary material for: Method Matters: Effect of Purification Technology on Neutrophil Phenotype and Function
Source: Front Immunol. 2022 Feb 10;13:820058. doi: 10.3389/fimmu.2022.820058 (PMC8866851; doi:10.3389/fimmu.2022.820058)
Supplement: Supplementary file 1 [file DataSheet_1.pdf]

Supplementary Material

Supplementary Figures

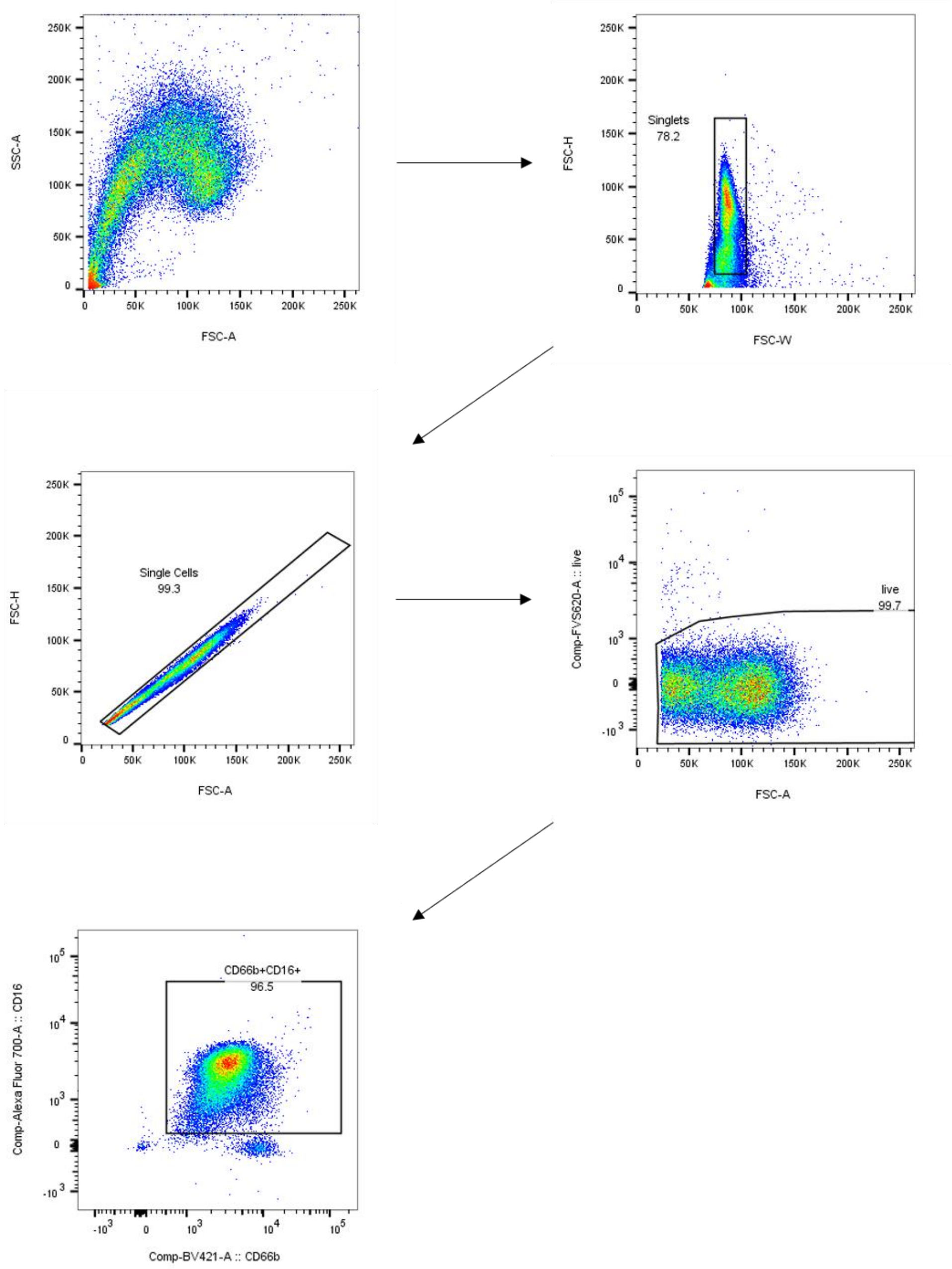

Supplementary Figure 1: Gating strategy for flow cytometry experiments. Gating strategy for flow cytometry analysis of purified peripheral blood neutrophils (neutrophils from density-gradient purification are shown as example). Single cells were gated based on forward scatter height, width and area (FSC-H, FSC-W, FSC-A). Dead cells were excluded by uptake of Fixable Viability Stain 620 (FVS620) or Zombie Aqua 516. Living, single cells were determined to be neutrophils if they expressed both CD16 and CD66b ($CD16^{+}CD66b^{+}$ cells).

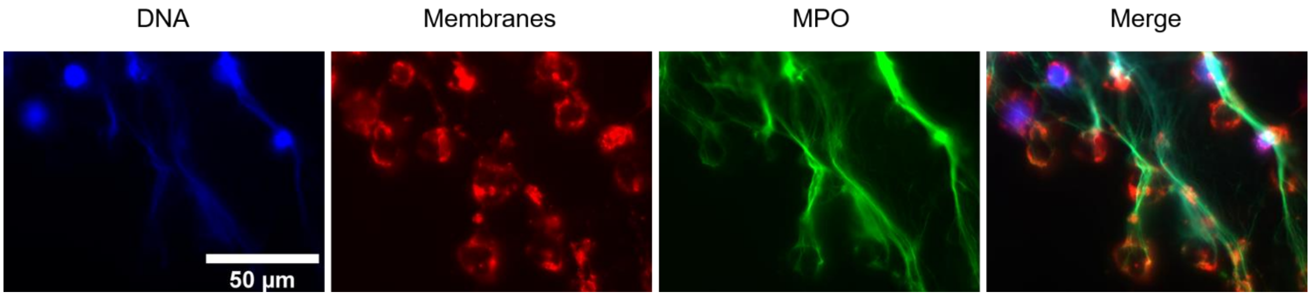

Supplementary Figure 2: Immunofluorescent co-staining of DNA and MPO showing NETosis of neutrophils. To confirm that the purified neutrophils released DNA due to NETosis, an immunofluorescent staining was performed. To this end, neutrophils were exposed to PMA (150 ng/ml) for 3 hours at 37°C. The cells were treated with polyclonal rabbit anti-human MPO antibody (MPO), Hoechst (DNA) and Alexa Fluor 594-conjugated wheat germ agglutinin (Membranes). A representative image of immunomagnetically purified neutrophils treated with PMA, is shown. Similar co-localization was seen for NETosing neutrophils in other conditions.

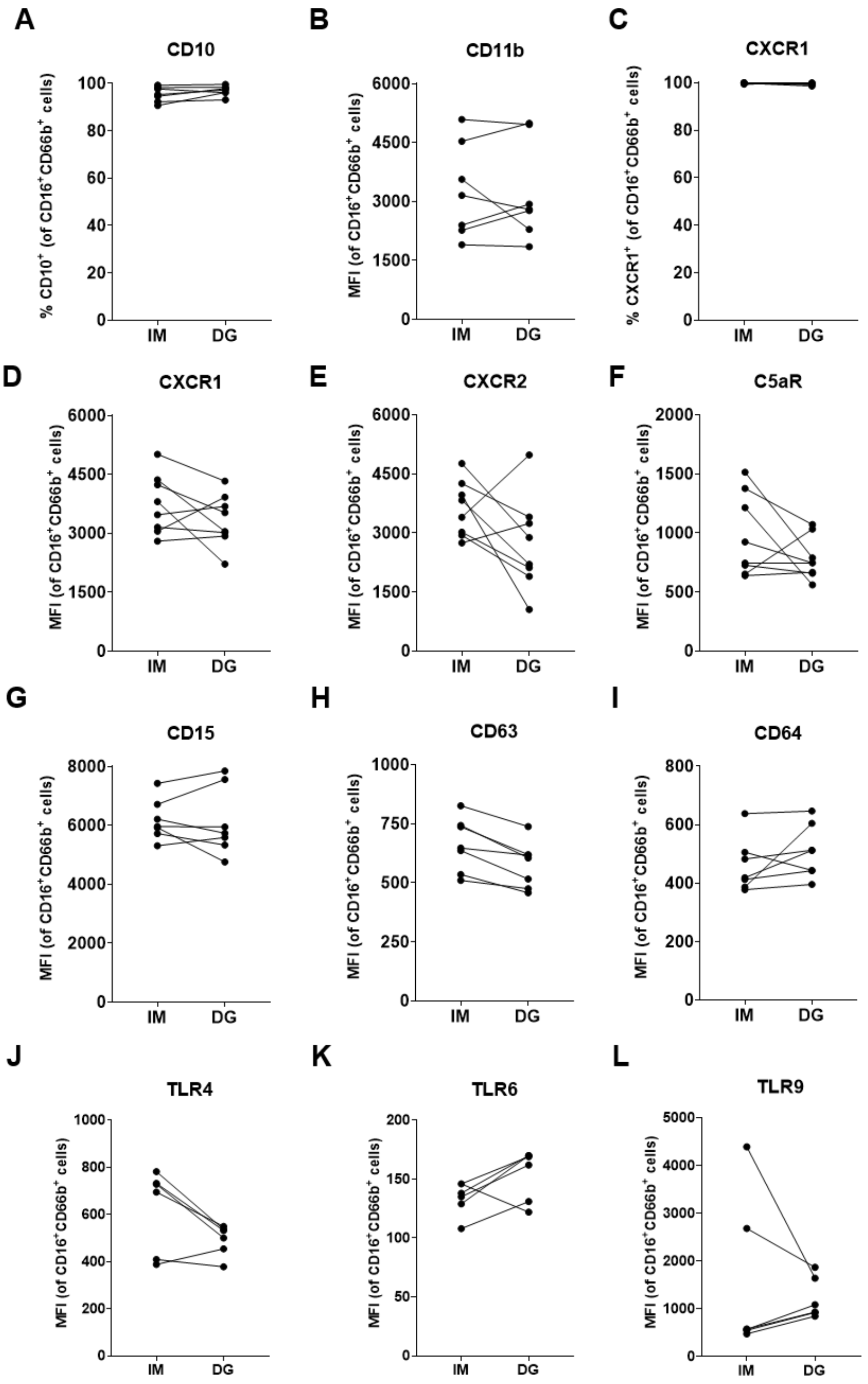

Supplementary Figure 3: Phenotypical characterization of neutrophils isolated by immunomagnetic or density-gradient purification. Flow cytometry was used to evaluate the surface expression of **(A)** CD10, **(B)** CD11b, **(C-D)** CXCR1, **(E)** CXCR2, **(F)** C5aR, **(G)** CD15, **(H)** CD63, **(I)** CD64, **(J)** TLR4, **(K)** TLR6 and **(L)** TLR9 on neutrophils (gated as $CD16^{+}CD66b^{+}$ cells) isolated by immunomagnetic (IM) or density-gradient purification (DG) from peripheral blood of healthy donors. Results are represented as percentage of neutrophils positive for the marker or median fluorescence intensity (MFI). Results from each individual donor (n = 6-8) are connected by lines and were statistically analyzed by Wilcoxon matched-pairs signed rank test.

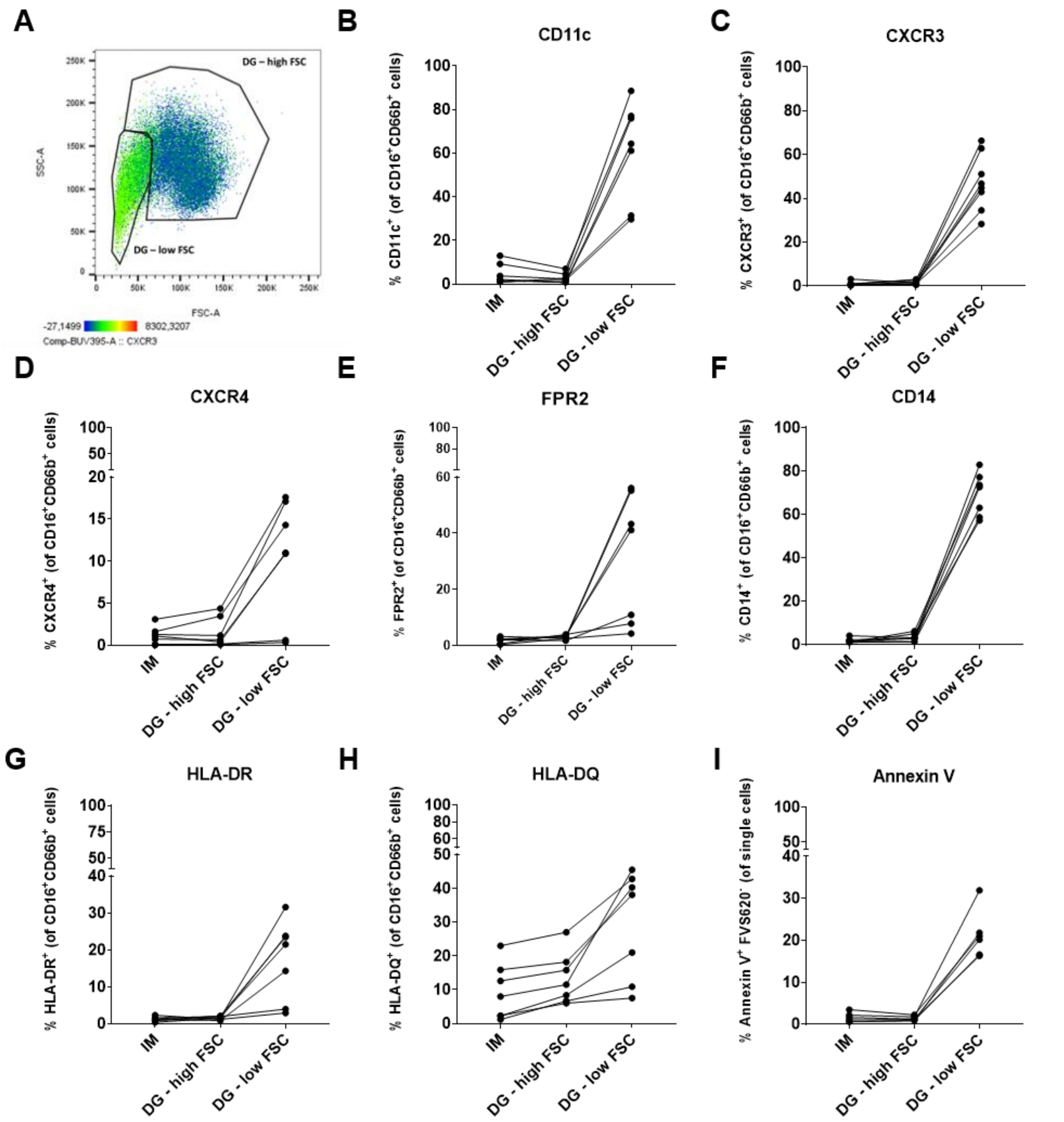

Supplementary Figure 4: Expression of atypical markers on immunomagnetic and density-gradient purified neutrophils, stratified by cell size. (A) Differentiation of neutrophils obtained by density gradient purification based on FSC/SSC gating. Flow cytometry was used to evaluate the surface expression of **(B)** CD11c, **(C)** CXCR3, **(D)** CXCR4, **(E)** FPR2, **(F)** CD14, **(G)** HLA-DR, **(H)** HLA-DQ and **(I)** Annexin V on neutrophils (gated as $CD16^{+}CD66b^{+}$ cells) isolated by immunomagnetic (IM) or density gradient purification (DG) from peripheral blood of healthy donors. Density-gradient-purified neutrophils were stratified in two populations based on cell size. Results are represented as percentage of neutrophils positive for the marker. Results from each individual donor (n = 6-8) are connected by lines.

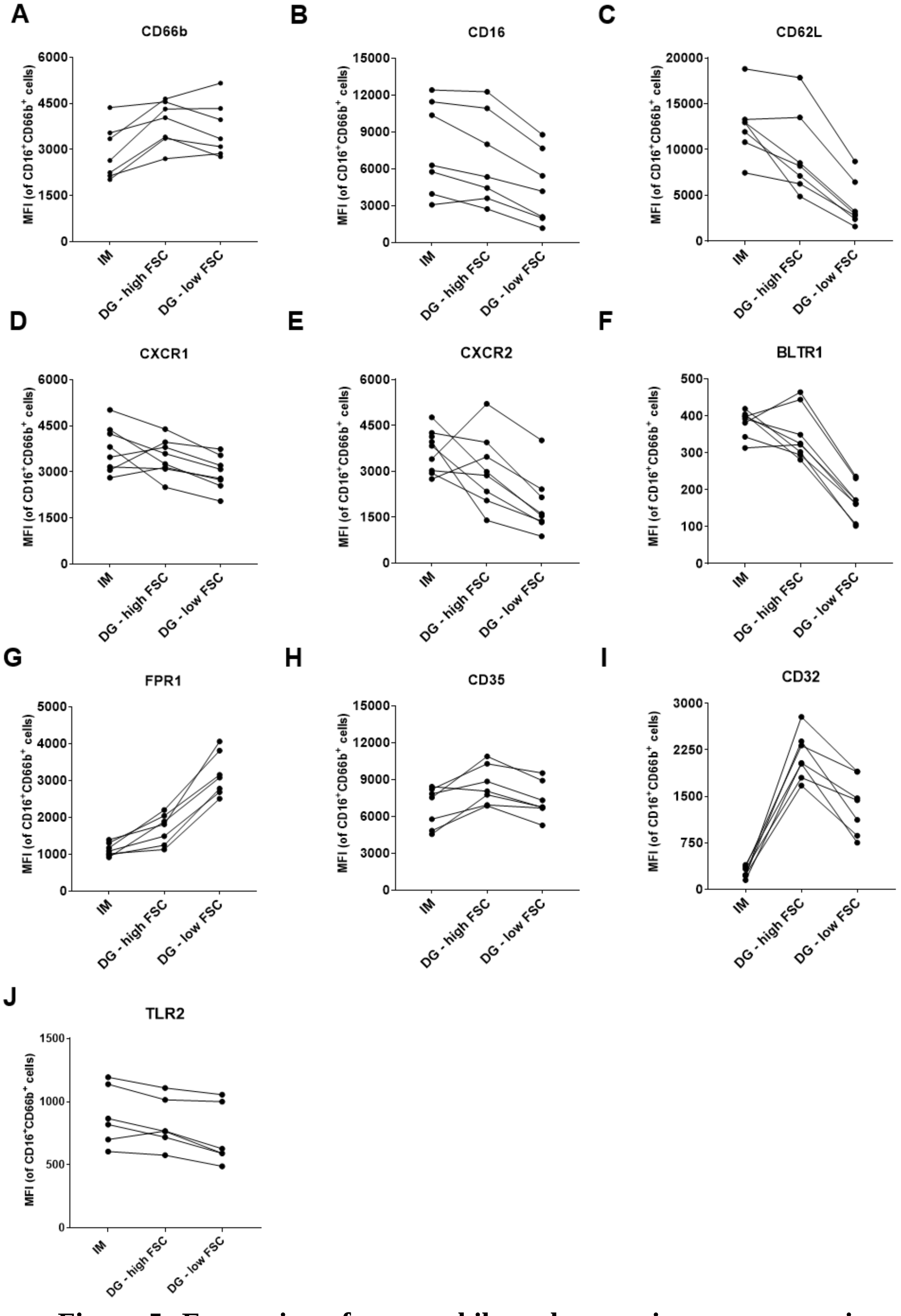

Supplementary Figure 5: Expression of neutrophil markers on immunomagnetic and density-gradient purified neutrophils, stratified by cell size. Flow cytometry was used to evaluate the surface expression of **(A)** CD66b, **(B)** CD16, **(C)** CD62L, **(D)** CXCR1, **(E)** CXCR2, **(F)** BLTR1, **(G)** FPR1, **(H)** CD35, **(I)** CD32 and **(J)** TLR2 on neutrophils (gated as $CD16^{+}CD66b^{+}$ cells) isolated by immunomagnetic (IM) or density-gradient purification (DG) from peripheral blood of healthy donors. Density-gradient-purified neutrophils were stratified in two populations based on cell size. Results are represented as median fluorescence intensity (MFI) of neutrophils. Results from each individual donor (n = 6-8) are connected by lines.

**Reagent**	**Clone**	**Label**	**Host species**	**Company**
Annexin V		BV711		BD Biosciences
Anti-CD10	HI10a	BV786	Mouse	BD Biosciences
Anti-CD11b	ICRF44	BV510	Mouse	BD Biosciences
Anti-CD11b	ICRF44	APC-Cy7	Mouse	Biolegend
Anti-CD11c	3.9	eFluor710	Mouse	eBioscience
Anti-CD14	61D3	PE-Cy7	Mouse	eBioscience
Anti-CD15	HI98	BUV395	Mouse	BD Biosciences
Anti-CD15	W6D3	BV786	Mouse	BD Biosciences
Anti-CD16	3G8	Alexa Fluor 700	Mouse	BD Biosciences
Anti-CD16	3G8	BUV395	Mouse	BD Biosciences
Anti-CD32	FLI8.26	BV711	Mouse	BD Biosciences
Anti-CD35	E11	FITC	Mouse	Biolegend
Anti-CD62L	DREG56	APC	Mouse	eBioscience
Anti-CD63	H5C6	BV510	Mouse	BD Biosciences
Anti-CD64	10.1	eFluor710	Mouse	Invitrogen
Anti-CD66b	G10F5	BV421	Mouse	BD Biosciences
Anti-CD66b	G10F5	PerCP-Cy5.5	Mouse	Biolegend
Anti-CD88 (C5aR)	S5/1	PerCP-Cy5.5	Mouse	Biolegend
Anti-CD181 (CXCR1)	5A12	PE	Mouse	BD Biosciences
Anti-CD182 (CXCR2)	6C6	FITC	Mouse	BD Biosciences
Anti-CD183 (CXCR3)	1C/CXCR3	BUV395	Mouse	BD Biosciences
Anti-CD184 (CXCR4)	12G5	BUV395	Mouse	BD Biosciences
Anti-CD282 (TLR2)	11G7	BV421	Mouse	BD Biosciences
Anti-CD284 (TLR4)	HTA125	FITC	Mouse	Abcam
Anti-CD286 (TLR6)	TLR6.127	PE	Mouse	Biolegend
Anti-CD289 (TLR9)	eB72-1665	APC	Mouse	eBioscience
Anti-BLTR1	203/14F11	BV510	Mouse	BD Biosciences
Anti-FPR1	5F1	Alexa Fluor 647	Mouse	BD Biosciences
Anti-FPR2	GM1D6	PE	Mouse	Santa Cruz
Anti-HLA-DQ	Tu169	Alexa Fluor 647	Mouse	BD Biosciences
Anti-HLA-DR	L243	BV650	Mouse	Biolegend

Supplementary Table 1. Overview of antibodies used for flow cytometry. APC, allophycocyanin; BUV, Brilliant Ultraviolet; BV, Brilliant Violet; FITC, fluorescein isothiocyanate; PE, phycoerythrin; PerCP, Peridinin Chlorophyll Protein Complex.
